# Hygiene and disinfection measures for parvovirus B19 infections

**DOI:** 10.3205/dgkh000534

**Published:** 2025-03-05

**Authors:** Maren Eggers, Nils-Olaf Hübner, Ulrike Helber-Soszynski, Johannes Blümel, Martin Exner, Jürgen Gebel, Carola Ilschner, Holger F. Rabenau, Ingeborg Schwebke, Martin Enders

**Affiliations:** 1 Association for Applied Hygiene (VAH), Bonn, Germany; 2 Virus Disinfection Commission of the German Association for the Control of Virus Diseases (DVV), Kiel, Germany; 3 Germany Society of Virology (GfV), Aschaffenburg, Germany; 4 Laboratory Prof. Gisela Enders MVZ, Stuttgart, Germany; 5 Institute of Hygiene and Environmental Medicine, University Medicine Greifswald, Germany; 6 German Society for General and Hospital Hygiene (DGKH), Berlin, Germany; 7 Paul-Ehrlich-Institut (Federal Institute for Vaccines and Biomedicines), Langen, Germany; 8 Institute of Hygiene and Public Health of Bonn University Hospital, Germany; 9 Institute for Medical Virology, Goethe-University Frankfurt, Germany; 10 German Consulting Laboratory for Parvoviruses, Stuttgart, Germany

**Keywords:** parvovirus B19, prenatal infection, hydrops fetalis, pregnancy, erythema infectiosum, anemia, virucidal PLUS, disinfection, hygiene, fifth disease, health care

## Abstract

**Background:**

In 2024, there has been a notable surge in the incidence of infections with parvovirus B19 (B19V). This development presents significant challenges, particularly for pregnant women, daycare centers, and medical facilities with vulnerable patients.

**Properties of B19V:**

B19V is transmitted primarily by droplet infection, directly and indirectly through contaminated surfaces. It is highly contagious and can have serious health consequences for at-risk groups, especially for pregnant women and immunocompromised individuals. There is not yet much data available on the specific tenacity/replication capacity of B19V, which is why empirical values from animal parvoviruses regarding sensitivity to disinfectants are used.

**Recommendations for hygiene measures:**

In order to prevent the further spread of B19V, an advisory has been prepared by experts from the Association for Applied Hygiene (VAH), in collaboration with the “Virus Disinfection” commission of the German Association for the Control of Virus Diseases (DVV) and the Society for Virology (GfV). This advisory is based on a risk assessment and the specific properties of B19V, and it outlines the necessary hygiene measures, including the selection of suitable disinfectants, to be taken in various areas in order to prevent the virus from spreading further. In the domestic setting, fundamental hygiene practices include thorough handwashing, refraining from touching the eyes, mouth, and nose, the use of a medical mask in the absence of physical distancing, and the frequent ventilation of rooms. In a medical setting, for example, the disinfection of surfaces in close proximity to the patient with an active virucidal agent and the use of gloves are also recommended when dealing with infected or potentially infected individuals.

## Zusammenfassung

**Hintergrund:**

Im Jahr 2024 war ein signifikanter Anstieg von Infektionen mit dem Parvovirus B19 (B19V) zu verzeichnen. Diese Entwicklung konfrontierte insbesondere Schwangere, Kindertagesstätten sowie medizinische Einrichtungen mit vulnerablen Patientinnen und Patienten mit beträchtlichen Herausforderungen.

**Eigenschaften des B19V:**

Das B19V wird vor allem durch Tröpfcheninfektion, direkt und indirekt über kontaminierte Oberflächen übertragen. Es weist eine hohe Ansteckungsfähigkeit auf und kann bei Risikogruppen, insbesondere bei Schwangeren und immungeschwächten Personen, zu gravierenden gesundheitlichen Konsequenzen führen. Zur spezifischen Tenazität des B19V liegen noch nicht viele Daten vor, weshalb Erfahrungswerte der animalen Parvoviren zur Empfindlichkeit gegenüber Desinfektionswirkstoffen herangezogen werden.

**Schlussfolgerungen für Hygienemaßnahmen:**

Vertreterinnen und Vertreter vom Verbund für angewandte Hygiene e.V. (VAH) zusammen mit der Kommission „Virusdesinfektion“ der Deutschen Vereinigung zur Bekämpfung der Viruskrankheiten e.V. (DVV) und der Gesellschaft für Virologie e.V. (GfV) haben auf der Grundlage einer Risikobewertung sowie der spezifischen Eigenschaften des B19V eine Übersicht über Hygienemaßnahmen, einschließlich geeigneter Desinfektionsmittel, in verschiedenen Bereichen erstellt, um eine weitere Ausbreitung des Virus zu verhindern. Die Basishygiene im Haushalt beinhaltet gründliches Händewaschen, Vermeidung des Berührens von Augen, Mund und Nase, Tragen einer medizinischen Maske, falls keine räumliche Trennung möglich ist, und häufiges Lüften. Im medizinischen Umfeld werden für den Umgang mit Infizierten und Infektionsverdächtigen beispielsweise auch die Desinfektion patientennaher Flächen mit dem Wirkungsbereich viruzid PLUS und das Tragen von Handschuhen empfohlen.

## Introduction

Parvovirus B19 (B19V) is the causative agent of **Erythema infectiosum**, an infectious disease that is usually harmless and occurs mainly in children (“fifth disease”, “slapped-cheek syndrome”). B19V is spread worldwide. Increased numbers of cases have been reported in Germany and Europe in 2024 [1]. In our view, the exceptionally high level of activity can be explained, among other things, by the fact that the number of cases was very low during the SARS-CoV-2 pandemic due to lockdowns and hygiene measures and that the virus *now* has a significantly higher number of susceptible hosts at its disposal. Irrespective of this, the periodic occurrence of epidemics at intervals of around 4-5 years can be observed [2]. As neither the virus nor the disease are notifiable according to the German Infection Protection Act (§§ 6, 7 IfSG [3]), there are no official figures on the current incidence of infection in Germany. However, in the case of clusters (two or more nosocomial infections), there is an obligation to notify the public health department, even for non-notifiable infections, in accordance with IfSG Section 6 (3) sentence 1.

### Characteristics of the virus

The non-enveloped parvoviruses belong to the family *Parvoviridae*, genus Erythroparvovirus and have a very compact structure. Parvoviruses exhibit a very high environmental stability due to their DNA, which is present as a linear, single-stranded molecule, and the lack of a lipid envelope. B19V replicates primarily in human erythroid bone marrow cells. The initial infection leads to a temporary arrest of hematopoiesis. The drop in hemoglobin is usually not clinically relevant in immunocompetent individuals without underlying hematological disease. 

### Transmission routes

Horizontal transmission occurs primarily through droplet infection (directly or indirectly via contaminated surfaces), for example when sneezing or coughing. Significant contact occurs after staying in the same room for at least 15 minutes or “face-to-face” contact with a laboratory-confirmed case during its period of highest infectivity (7 days before the onset of exanthema until the onset of exanthema) [4]. The extent to which there is residual infectivity after the onset of the "typical" symptoms (rash, arthropathies) is unclear.

Transmission via blood or plasma donations is very rare, but is possible as a result of pronounced viraemia (up to 10^13^ particles/ml) during the incubation period. In many places, highly viremic donations are now sorted out on a voluntary basis [2].

Nosocomial transmissions have also been documented in the literature. However, the risk of nosocomial transmission has been discussed controversially [5], [6], [7], [8]. Riipinen et al. [9] found a 2.6-fold increased risk of B19V infection in kindergarten and daycare center employees compared to women working in the healthcare sector.

### Replication capacity on inanimate surfaces and inactivation

Specific data on the replication capacity and inactivation of B19V are scarce [10]. Animal parvoviruses such as the minute virus of mice (MVM), porcine (PPV), canine (CPV) or bovine parvovirus (BPV) retain their infectivity when dried. They are heat-resistant up to approx. 90°C and stable even at low pH values (up to pH <2). In contrast to these animal parvoviruses, B19V has been shown to be inactivated at temperatures as low as 60°C [11]. In addition, B19V is inactivated at pH 4 [12] and is sensitive to guanidine hydrochloride [13]. Therefore the susceptibility of B19V to some disinfection methods/agents may differ from that of animal parvoviruses such as MVM or the adeno-associated virus (AAV) frequently used in gene therapy. 

### Incidences of infections

The incidence of erythema infectiosum is highest in kindergarten, preschool and primary school children. Household contacts harbor the greatest risk of acquiring a B19V infection (approx. 50% of susceptible people become infected) [14], [15], [16]. The risk of acquiring an infection in the context of a B19V outbreak in an institution was reported to be 31% for (day-care) personnel with contact to younger children and 23% for primary school teachers in a US publication in JAMA from 1990 [17]. 

### Incubation period

The time between exposure and viraemia and the associated infectivity is typically 5–14 days [18], [19]. The incubation period until the onset of classic symptoms (exanthema, joint pain) is 7–18 (up to 21) days. They are often preceded by unspecific prodromal symptoms. 

### Clinical signs and symptoms

The exanthema usually begins in childhood with a mouth omission and a conspicuous reddening of the cheeks (“slapped cheek syndrome”) as well as a rash on the arms and legs. This can be present in various forms or be completely absent. It is characterized by a reticular or lacy appearance during the course of the disease. Childhood B19V infections are usually without complications. Diseases in adulthood occasionally show prolonged convalescence phases. 

In the immunocompetent host without underlying disease B19V infections usually proceed without complications. Rarely severe disease such as pancytopenia, meningitis/encephalitis/encephalopathy, myocarditis/cardiomyopathy, hepatitis, and glomerulonephritis has been associated with B19V infection. But a causal relationship is not always established. Certain patient groups or populations are at an increased risk for B19V-derived complications: e.g. aplastic crisis in people with impaired hematopoiesis, chronic anemia with immunosuppression, abortion/fetal anemia/hydrops fetalis during pregnancy [20]. 

As signs and symptoms vary and infections are often asymptomatic, an acute B19V infection can ultimately only be reliably confirmed or ruled out with appropriate laboratory tests (detection of specific IgG and IgM antibodies, quantitative determination of B19V DNA using NAT, possibly determination of IgG avidity or epitope-specific IgG antibodies, e.g. using immunoblotting) [21], [22], [23], [24]. 

The immunity acquired through an infection generally leads to lifelong protection in immunocompetent individuals. Reinfections are likely to occur, but are rare [25]. To date, there is no evidence that reinfections lead to complications in immunocompetent individuals.

### Parvovirus B19 infections during pregnancy

The B19V seroprevalence in pregnant women or women of childbearing age is between 60% and 70% [14], [15], [26]. This means that approximately one in three women does not have protection against infection with B19V. Ideally, testing for B19V antibodies should be carried out before or as soon as possible after determining the pregnancy. However, costs for diagnosis of B19V infection during pregnancy are covered by the German statutory health insurance only in the event of contact with an infected person, maternal symptoms (e.g., rash) or the presence of abnormal ultrasound findings. In other situations determination of the B19V immune status is offered as an individual health service (IGeL). In other countries, other rules and regulations may apply. The information for Germany was retrieved on 19 September 2024 from the German Federal Ministry of Health website https://gesund.bund.de/ringelroeteln?pk_campaign=ghp).

In around 30–50% of cases, an infection is asymptomatic, even in pregnant women. However, the virus can also be transmitted to the fetus in asymptomatic cases. The intrauterine transmission risk is approx. 30–50% in the case of initial maternal infection in the 2^nd^ trimester (gestational week 14+0 – gestational week 27+6) and increases in the 3^rd^ trimester [27], [28]. The evidence on the risk of transmission after maternal infection in the 1^st^ trimester is limited. The most common complications of B19V infection in pregnancy are intrauterine fetal death (IUFT), fetal anemia and hydrops [29], [30], especially in infections in the first half of pregnancy (risk <10% in relation to maternal infection). Fetal complications occur in >90% of cases in the first 8 weeks after maternal infection. If the infection occurs after the 20^th^ week of pregnancy, the risk of severe fetal anemia or hydrops fetalis is very low. If an acute B19V infection is diagnosed during pregnancy, regular ultrasound and Doppler scans are recommended until 12 weeks after the onset of the infection [31]. Type and frequency of antenatal follow-up essentially depends on the gestational age at diagnosis of maternal infection. Invasive prenatal diagnosis and treatment (intauterine transfusion) should be considered in the presence of abnormal ultrasound or Doppler findings. The results of various observational studies suggest that timely intrauterine transfusion can significantly reduce the risk of fetal mortality [32], [33]. For fetuses with severe anemia and hydrops fetalis that have been successfully treated by intrauterine transfusion(s), an increased risk of neurodevelopmental delay is discussed [34], [35]. If hydrops fetalis is present in a newborn or stillborn infant with prenatal B19V infection, the child must be considered infectious [7]. 

### Therapy

There is currently no antiviral therapy or active vaccination against B19V. Patients with severe anemia or aplastic crisis can be treated with blood transfusions. In immunosuppressed patients with chronic anemia, treatment with immunoglobulin preparations, which usually contain a high concentration of B19V-specific antibodies, can be considered. If possible, immunosuppressive therapy should also be changed or reduced [36], [37]. 

## Preventive measures [42]

### Basic measures in the event of an outbreak in community facilities

The public health department can be called in to advise on hygiene measures. Pregnant women must not work during an outbreak. In the case of an immunocompromised child, the pediatrician in charge must decide and advise on how to proceed.

### Hand disinfection and hand hygiene

B19V is a non-enveloped virus with high intrinsic tolerance to alcohols, meaning that alcohol-based hand sanitizers (hand rubs) are not effective. For hand hygiene, it is therefore only possible to refer to wearing protective gloves and washing hands with soap combined with a hygienic hand disinfection, as recommended by the German Commission for Hospital Hygiene and Infection Prevention (KRINKO), e.g. for *C. difficile* [38].

### Surface disinfection

In the absence of specific data on the inactivation of B19V by disinfectants, it currently seems advisable to refer to experience with the animal model viruses.

The Virus Disinfection Commission of the German Association for the Control of Virus Diseases (DVV), the Society of Virology (GfV), and the Association for Applied Hygiene (VAH) have introduced the new “virucidal activity PLUS” efficacy range for disinfectants which prevent the transmission of particularly disinfectant-tolerant viruses such as HAV, HEV, *Parvoviridae* (such as B19V) [39]. For this purpose, the products are tested in a quantitative suspension test against adenovirus, norovirus, poliovirus and the SV40 virus and in a practical test without mechanics against adenovirus and norovirus and additionally against murine parvovirus (MVM). CAVE, the “virucidal” efficacy range (“virucidal”, also according to the current EN standards) for surface disinfection does not include efficacy against B19V.

For some products, a claim of “virucidal PLUS” for surface disinfection without mechanical action was confirmed by proving efficacy against the MVM in a simulated-use test [39]. It is possible to apply for VAH-certification of this activity spectrum and inclusion in the VAH Disinfectant List https://www.vah-liste.de/en/. Products for which efficacy has been demonstrated have peracetic acid, aldehydes, oxygen releasers or chloramine-T as their active ingredient base.

### Instrument disinfection

Semi-critical medical devices must be reprocessed using the virucidal efficacy spectrum in accordance with the recommendation of the Commission for Hospital Hygiene and Infection Control (KRINKO) and the Federal Institute for Drugs and Medical Devices (BfArM) [40].

For chemothermal instrument disinfection (>40°C), testing against the MVM is required to claim the “virucidal” efficacy range. Virucidal disinfectants for chemothermal instrument disinfection procedures (>40°C) are therefore effective against B19V. 

Testing against the MVM is not mandatory for chemical instrument disinfection procedures (<40°C). Therefore, in the event of a B19V outbreak, disinfectants must be used that have also been tested for parvovirus efficacy in a simulated-use test (test virus MVM). The same requirements for effectiveness against parvovirus also apply to manual instrument disinfection in the event of a B19V outbreak as for automated reprocessing.

### Textile disinfection

For the chemothermal disinfection of laundry, testing against MVM is also included as part of the “virucidal” claim. Therefore, virucidal laundry disinfectants tested accordingly are also effective against B19V. VAH-certified virucidal chemothermal washing processes are effective against B19V.

When washing in household washing machines, virus depletion can be assumed due to the temperature and the rinsing effect, although the extent is uncertain: B19V is inactivated at temperatures above 60°C [10]. Washing cycles from 60°C with a heavy-duty detergent therefore seem advisable. However, a household washing machine is no substitute for a disinfecting washing process.

### General hygiene and barrier measures

Preventing the exposure to *parvovirus* B19V prophylaxis is difficult, as infectivity exists before the usual symptoms (exanthema, arthropathy) appear and many infections are asymptomatic (clarify antibody status if necessary). Infectivity decreases significantly once the exanthema appears. However, residual infectivity may persist.

Exposure prophylaxis for people at risk in the household when non-immune pregnant women, people with immunodeficiency and patients with anemia come into contact with people with or suspected of having erythema infectiosum:

- Exposure prophylaxis should be limited to people at risk (non-immune pregnant women, especially before the 20^th^ week of pregnancy, people with immunodeficiency and patients with haematopoietic disorders, etc.) and, if applicable, their direct contacts.
- If possible, contact with people with known exposure should be avoided during the incubation phase.
- Once the symptoms have appeared in one’s own child, caring for the child by another person is no longer effective as a preventative measure.
- Communities/communal facilities with known erythema infectiosum infections should be avoided.

Standards precautions supplemented by measures against droplet transmission can reduce the likelihood of transmission:

- Wash your hands thoroughly and frequently.
- Avoid touching unwashed hands on the face (especially the nose, mouth and eyes).
- Wear a medical mask if physical distancing is not possible.
- Ensure frequent ventilation.

### Occupational health and safety when working with children and young people

The B19V immune status must be determined at the latest when the pregnancy becomes known. Seronegative pregnant women should be counselled on the risk (infection routes, risks for mother and fetus) and possible hygiene measures (e.g. frequent hand washing). Pregnant women who work in pre-school childcare centers can, in principle, be employed in “non-risk areas”. If this is not an option, in Germany, a temporary ban on employment is imposed until the 20^th^ week of gestation for seronegative women. Furthermore, a temporary ban on employment is issued after the 20^th^ week of gestation in instances where illnesses are reported in the work environment, continuing until 21 days after the most recent case.

In principle, there is no general ban on work or employment for people infected with B19V in communal facilities, as the risk of infection decreases rapidly once symptoms appear. If necessary, the person can wear a face mask for a limited period after resuming work. 

### Hygiene and barrier measures in the medical environment

The following general measures are recommended [15]:

- Regulations on erythema infectiosum should be included in the hygiene policy and be part of the risk assessment to be carried out by the employer.
- Infected persons or persons suspected of being infected should not visit patients in hospital. Patients and visitors should be informed of this.

When dealing with infected persons and suspected infections, the following should be noted:

- Isolate in accordance with hospital hygiene regulations and always in areas with immunocompromised patients, pregnant women and children.
- Strictly implement standard precautions (hand hygiene such as hand washing and gloves, avoidance of coughing, sneezing, preferably into the crook of the elbow, not coughing/sneezing into the hand) and adding measures to interrupt droplet/aerosol transmission (wearing a medical mask by staff and by patients when leaving the room, ventilating the room regularly).
- Wearing gloves can reduce hand contamination, followed by hand disinfection and subsequent washing.
- Use and safely dispose of disposable wipes.
- Disinfect surfaces close to patients at least daily with a disinfectant with the "virucidal PLUS" activity spectrum as included in the VAH List of Disinfectants (https://www.vah-liste.de/en/).
- Do not share use of towels, cutlery, crockery etc.
- Handle laundry in accordance with the rules for the transport and preparation of infectious laundry.
- Dispose of waste in accordance with the local hazardous waste regulations (e.g. AS 180104 (German disposal regulations)).
- Terminal disinfection after patient discharge.
- Additional information for contact persons: If possible, avoid surgery during the incubation period if the B19V immune status is unknown in order to avoid an aplastic crisis.
- Avoid contact with pregnant staff during breaks.
- Non-immune, exposed personnel (e.g. own child with fifth disease) should wear a medical mask and, if possible, be moved to areas without risk patients (in case of known contact with infected persons for 15–21 days).

When dealing with patients at risk of complications, the following should be noted:

- Avoid contact with people who are infected or suspected of being infected.
- Reduce contacts, no buffet supply.
- Inform patients.
- Employ standard precautions.
- Reverse isolation, if necessary.
- In pediatric oncology and hematology facilities especially in times of increased incidence, the B19V immune status of staff with direct/indirect patient contact should be known.

For immunocompromised patients, the following should be noted:

- Isolate patients with an aplastic crisis in a single room for 7 days after the onset of the illness.
- In addition to consistent implementation of the usual standard precaution measures, protect staff with PPE (protective gown, disposable gloves, surgical MNS); wearing protective goggles is also recommended for face-to-face contact or aerosol-generating measures.

Immunocompromised patients with chronic infections should always be considered potentially infectious [41]. 

## Notes

This recommendation has been published in German language [42].

### Authors’ ORCID

- Maren Eggers: 0000-0001-8485-9485
- Nils-Olaf Hübner: 0000-0002-6095-4936
- Carola Ilschner: 0009-0006-4083-7405
- Holger F. Rabenau: 0000-0001-9394-2176
- Ingeborg Schwebke: 0009-0007-6708-5845
- Martin Enders: 0000-0003-3218-074X

### Funding

Not applicable.

### Acknowledgments

Not applicable.

### Competing interests

The authors declare that they have neither financial nor non-financial competing interests.
